# Efficacy of Individual Computer-Based Auditory Training for People with Hearing Loss: A Systematic Review of the Evidence

**DOI:** 10.1371/journal.pone.0062836

**Published:** 2013-05-10

**Authors:** Helen Henshaw, Melanie A. Ferguson

**Affiliations:** NIHR Nottingham Hearing Biomedical Research Unit, Nottingham, United Kingdom; UNLV, United States of America

## Abstract

**Background:**

Auditory training involves active listening to auditory stimuli and aims to improve performance in auditory tasks. As such, auditory training is a potential intervention for the management of people with hearing loss.

**Objective:**

This systematic review (PROSPERO 2011: CRD42011001406) evaluated the published evidence-base for the efficacy of individual computer-based auditory training to improve speech intelligibility, cognition and communication abilities in adults with hearing loss, with or without hearing aids or cochlear implants.

**Methods:**

A systematic search of eight databases and key journals identified 229 articles published since 1996, 13 of which met the inclusion criteria. Data were independently extracted and reviewed by the two authors. Study quality was assessed using ten pre-defined scientific and intervention-specific measures.

**Results:**

Auditory training resulted in improved performance for trained tasks in 9/10 articles that reported on-task outcomes. Although significant generalisation of learning was shown to untrained measures of speech intelligibility (11/13 articles), cognition (1/1 articles) and self-reported hearing abilities (1/2 articles), improvements were small and not robust. Where reported, compliance with computer-based auditory training was high, and retention of learning was shown at post-training follow-ups. Published evidence was of very-low to moderate study quality.

**Conclusions:**

Our findings demonstrate that published evidence for the efficacy of individual computer-based auditory training for adults with hearing loss is not robust and therefore cannot be reliably used to guide intervention at this time. We identify a need for high-quality evidence to further examine the efficacy of computer-based auditory training for people with hearing loss.

## Introduction

### Background

The World Health Organization [1] reported in 2004 that over 275 million people worldwide had a significant hearing impairment. Adult-onset hearing loss is highly prevalent, whereby 27% of males and 24% of females aged 45 years and over experience mild hearing loss (defined as a pure-tone hearing threshold of 26 decibels (dB) average across 0.5, 1, 2, 4 k Hz) or greater in the better hearing ear. Hearing loss is currently estimated to be the 13^th^ most common disease burden worldwide, and it has been predicted that by 2030 adult-onset hearing loss will be the seventh leading disease burden, above diabetes and HIV [1]. Hearing loss can lead to additional difficulties with employment, depression, social isolation, and reduced quality of life [2]. Untreated hearing loss has a substantial social impact on the person with hearing loss and for those with whom they communicate [3], [4].

For adults who gradually acquire a hearing loss, their first complaint is unlikely to be ‘I cannot hear’. More often they report ‘I can hear but I cannot understand what is being said’, particularly in noisy listening environments [5]. Hearing aids are the most common management option for people with hearing loss, yet uptake is low, with just 20% of people with hearing loss in the UK [6], [7], and just under 30% in the US [8] owning them. Furthermore, of those who do own hearing aids, between 15% and 30% do not wear them regularly [6], [9]. It has become apparent over the last decade that the challenges faced by people with hearing loss cannot be explained by the audiogram alone [5], [10]. Difficulties in hearing may be exacerbated by, or masquerade as, reductions in cognitive ability such as problems in remembering or comprehending speech [11], [12]. Although hearing aids may help people with hearing loss *hear* speech, their ability to *listen to* and *make sense of* speech may still be sub-optimal. Cognitive function plays a significant role in listening, whereby greater working memory capacity is associated with improved language comprehension [13], and selective attention has been shown to be central to following multi-speaker conversations (see [14] for a review).

The 2012 British Society of Audiology guidance for adult hearing rehabilitation [15] states that successful rehabilitation should be based upon, ‘identifying individual needs, setting specific goals, making shared informed decisions and supporting self-management – steps that are important for helping the client to overcome his/her difficulties in everyday life’ [15]. To enable this, clinicians may need to consider interventions that are complementary or alternative to the provision of hearing aids (or cochlear implants where hearing loss is severe to profound). Auditory training is one such clinical intervention, which promotes self-management of hearing difficulties, and is aimed at improving speech intelligibility through the development of auditory perceptual skills. Typically, listeners learn to make perceptual distinctions between sounds presented systematically [16]. Studies of auditory perceptual learning demonstrate the potential for training to improve auditory perceptual skills over the course of an adult’s lifespan (see [17] for a review).

### Auditory Training as an Intervention for People with Hearing Loss

Historically, a distinction has been made between bottom-up sensory refinement (analytic training) and top-down improvement of spoken language comprehension (synthetic training) [18]. In 2005, Sweetow and Palmer published a systematic review of studies that assessed the efficacy of individual auditory training for adults with hearing loss [18]. These studies assessed clinician-delivered training, an intervention which is time-, resource-, and cost-intensive. Six articles, published between 1970 and 1996 [19]–[24], met the criteria for inclusion, which were; *Participants:* adults with hearing loss with or without hearing aids, who were not cochlear implant users; *Intervention*: analytic or synthetic auditory training, or combination of the two; *Controls:* with or without a control group comparison; *Outcomes*; one or more measure relating to communication skills (e.g. understanding speech, self-perception of ability); *Study designs:* randomized controlled trials, nonrandomized controlled trials, cohort and repeated measures designs with or without a control group. The authors concluded that speech recognition skills, particularly in noise, may be improved by synthetic training, whereas the contribution of analytic training remains uncertain. Yet, more recently, bottom-up (analytic) auditory training using phoneme discrimination has also shown improvements in top-down cognitive processing, which may offer additional benefit to people with hearing loss, particularly in adverse listening situations [25]. Finally, a meta-analysis of six studies assessing the benefits of (primarily clinician-delivered) auditory training for people with hearing loss published between 1970 and 2009, (including those reviewed by Sweetow & Palmer [18]), suggested a reliable but small post-training improvements in speech recognition performance (Cohen’s *d* = .352) [26].

Over the last two decades, the emergence of individual (non-clinician delivered), computer-based auditory training (CBAT) packages has resulted in a resurgence of interest in auditory training as an intervention for people with hearing loss. The key benefits of CBAT include home-delivery, the potential to tailor training packages to individual needs, and the ability to remotely monitor and capture trainee data over the internet. Thus, CBAT is an intervention that is time-, resource- and cost-effective, and can be conveniently accessed by the user [27]. There are several key considerations in using individual CBAT as an intervention to improve speech intelligibility for people with hearing loss. First and foremost, the intervention should be demonstrated to be effective, whereby any on-task learning should generalise to functional benefits in real-world listening ability. Improvements in behavioural measures of speech intelligibility in noise are typically considered by researchers and clinicians to be the ultimate aim of CBAT, as this is the most common complaint of people with hearing loss. However, as speech intelligibility has been shown to be mediated by cognition, particularly where the speech signal is degraded, training-related improvements in cognition (e.g. attention and working memory) are also likely to reflect functional real-world benefits to listening. Second, for auditory training to be accepted by an individual and therefore undertaken, the individual must be able to identify tangible benefits from the training. Thus, improvements in self-reported communication abilities are also important to the success of CBAT. Nevertheless, evidence from studies of alternative interventions suggest that improvements in self-reported outcomes alone may simply reflect expectations created as a result of receiving an intervention [28], [29]. Ideally, any improvements in self-reported outcomes should therefore be accompanied by functional benefits, as indexed by behavioural tasks of speech intelligibility or cognitive performance. Third, for auditory training to be a successful intervention for people with hearing loss, any CBAT related improvements should persist over time. Finally, individuals must comply with CBAT, as an intervention can only be effective when individuals conform. This final point is of particular importance where CBAT is used as an unsupervised, home-based intervention.

### Research Aims

A systematic review aims to identify, appraise and synthesize all the empirical evidence that meets pre-specified eligibility criteria in order to answer a given research question [30]. The primary aim of the present review was to examine the evidence for individual CBAT as an effective intervention for people with hearing loss. The evidence-base in the published literature was evaluated for both on-task learning for trained stimuli, and generalisation of learning to improvements in untrained measures of speech intelligibility, cognition and communication. Secondary aims sought to examine the feasibility of individual CBAT as an intervention for people with hearing loss by examining (i) the long-term retention of training-related improvements, and (ii) levels of compliance with CBAT programmes.

### Specific Research Questions

1. Does evidence exist to support improvements in trained and untrained measures of speech intelligibility, cognition or communication (either behavioural or self-reported outcomes) as a result of individual computer-based auditory training (CBAT) in people with hearing loss?
2. Do any improvement(s) in communication, speech intelligibility or cognitive abilities remain for people with hearing loss after CBAT has ceased (retention of learning)?
3. What are the levels of compliance with individual CBAT?

To address these questions, data from 13 published articles that met the criteria for inclusion were reviewed and quality assessed. Evidence for the efficacy of CBAT for people with hearing loss was extracted from included articles and the quality of evidence examined. Findings are presented together with recommendations for future research.

## Methods

The Centre for Reviews and Dissemination, University of York [31], part of the National Institute for Health Research (NIHR), and the Preferred Reporting Items for Systematic Reviews and Meta-analyses (PRISMA) statement [32], which offer guidance for undertaking and reporting of systematic reviews in health care, were used to inform the methodology, the systematic search procedure, and the reporting of this systematic review (Checklist S1).

### Systematic Search Strategy and Study Selection

Methods of data extraction, data analysis, and inclusion and exclusion criteria were pre-specified and documented within the systematic review protocol. This is important in providing transparency in the review process by ensuring that the objectives of the systematic review and methods of data identification and extraction are clearly defined prior to any data being collected. Details of the systematic review protocol have been registered with PROSPERO, the International prospective register of systematic reviews. The protocol is available online at: http://www.crd.york.ac.uk/prospero/display_record.asp?ID=CRD42011001406. Inclusion criteria were formed using the *Participants, Intervention, Control, Outcomes,* and *Study designs* (PICOS) strategy [33]. PICOS inclusion criteria are presented in Table 1. Exclusion criteria included articles that were published prior to 1996 (i.e. those included in the previous review of auditory training for people with hearing loss by Sweetow and Palmer, [18]), studies presenting pilot data, studies that were not peer reviewed and those not available in English.

**Table 1:** PICOS criteria for inclusion.

**P**articipants	Adult (18+ years) humans with any degree of hearing loss
**I**ntervention	Individual computer-based auditory training.
**C**ontrol	Comparison with a control group or repeated measures [pre- and post-training comparison].
**O**utcomes	1+ outcome measure(s) related to speech intelligibility, cognition or communication (either behavioural measures or self-reported outcomes).
**S**tudy designs	Randomised controlled trials, non-randomised controlled trials, cohort studies (with a control comparison), and repeated measures (pre- and post-training comparisons).

#### Study identification

Eight electronic databases (Embase, Medline, Pubmed, Web of Science, Applied Social Sciences Index and Abstracts (ASSIA), Science Direct,/Cumulative Index to Nursing and Allied Health (CINAHL) and PsychINFO) were initially searched in August 2011 using the terms hearing loss OR hearing aid* OR hearing impair* OR cochlear implant* AND auditory training OR auditory learning OR perceptual training OR perceptual learning. Search terms were always combined in an attempt to limit identified papers to those reporting adult subjects with hearing loss. An example search string is provided (Example Search Terms S1). Additional articles were identified through the systematic snowballing of all 349 articles reference lists, and a related article search for each author of an article which met the PICOS criteria for inclusion. Three further articles were identified through ongoing hand-searches of audiology journals, up to the date of first submission of this article (December 2012), to ensure an up-to-date review.

#### Screening

**Figure 1:**
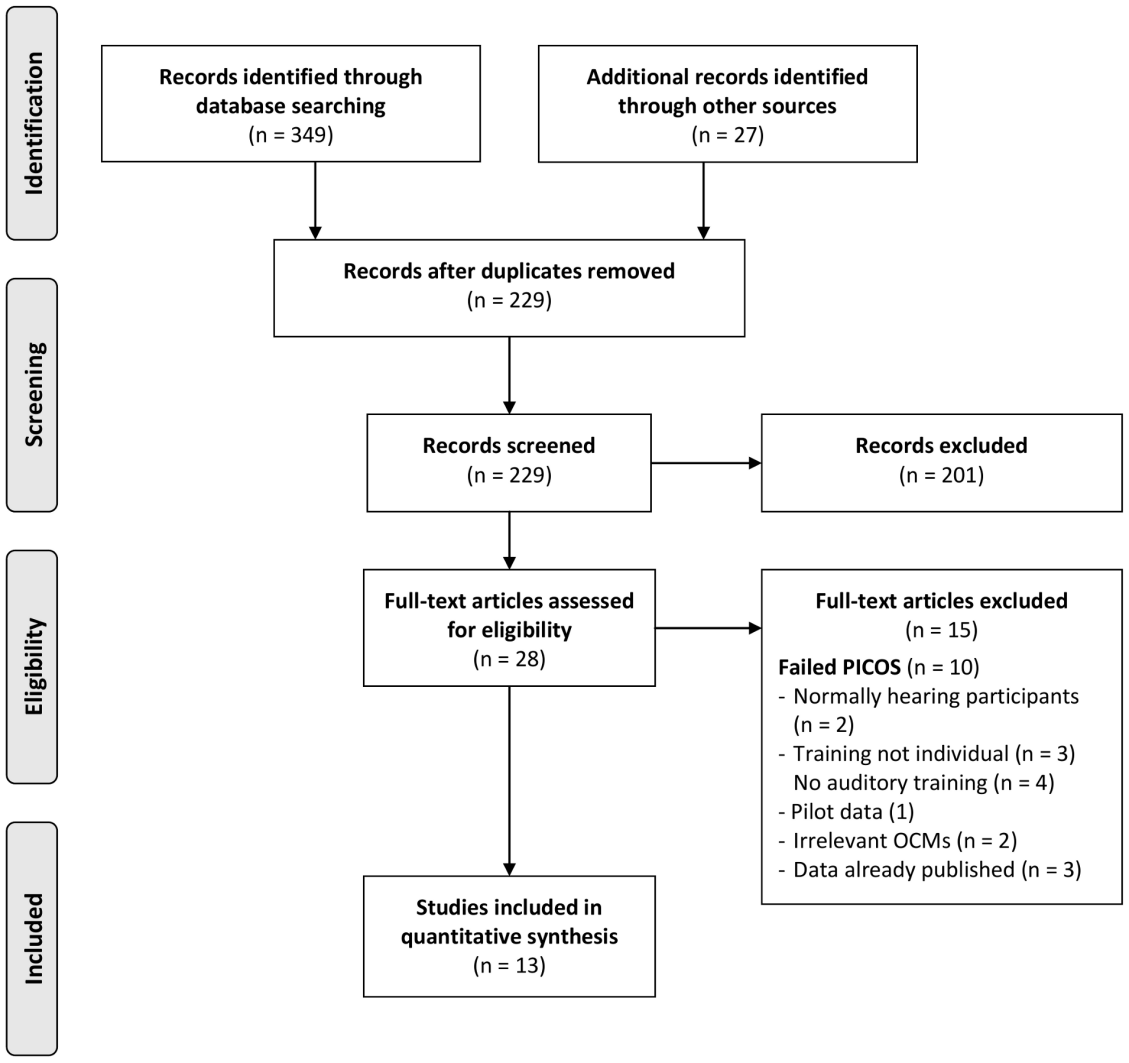
Flow diagram of the study identification, eligibility, and inclusion process.

The database searches returned a total of 349 articles. A further 27 articles were identified through the additional journal searches. A total of 229 articles of potential relevance remained after the removal of duplicate articles (n = 147). Abstracts of the 229 identified articles were independently assessed by the two authors relative to the PICOS criteria for inclusion (Figure 1), of which, 201 failed to meet the inclusion criteria. In cases where insufficient detail was available in the abstract to make a decision, the full-text of the article was retrieved and assessed against the PICOS criteria. A total of 28 abstracts either met the PICOS criteria for inclusion, or contained insufficient information from which to make a judgement, and progressed to a second stage of screening where the full-text articles were obtained.

#### Eligibility

A second stage of assessment, a full-text review of the 28 potentially relevant articles, revealed 15 articles that failed to meet the PICOS criteria for inclusion. For cases where multiple publications arose from the same participant data, only the first publication was included in line with the Centre for Reviews and Dissemination guidelines [31]. A total of 13 articles were eligible for inclusion in the systematic review.

### Data Extraction and Data Synthesis

Data to be extracted were pre-specified within a data extraction and quality assessment form, piloted by the two authors and amended as necessary. Final study data extraction was conducted independently by the same two authors and included details of study design, participants (number, age, sex and hearing loss), training stimuli, amount of training and training duration, outcome measures, main findings (both trained and untrained), compliance and follow-up. Where any instances of non-agreement on the extracted data occurred, the article was jointly revisited until a consensus was reached.

### Study Quality and Potential Sources of Study Bias

Scientific study quality and potential sources of study bias were assessed using five independent measures; randomisation, controls, sample size and power calculation, blinding, and outcome measure reporting. Low scores on these measures indicate less information, thus a higher potential for bias in results. Five additional measures, which were all highly specific to training intervention studies, aimed to capture the quality of the intervention study designs. Measures included; generalisation of learning to functional benefits in real-world listening (outcome selection), training feedback, which has been previously shown to maximise auditory learning in auditory training [33], [34], ecological validity, compliance with training protocols, and long-term follow-up of improvements. Scores for each of the study quality measures ranged from 0–2. A score of 0 indicated flawed or no information from which to make a judgement, a score of 1 indicated weak information or lack of detail and a score of 2 indicated appropriate use and reporting.

Individual measure scores were summed to form an overall study quality score that was then used to inform the level of evidence attributed to each study. Levels of evidence were adapted from the 2004 Grading of Recommendations Assessment, Development and Evaluation (GRADE) Working Group guidelines [35] and provide an indication of confidence in the estimation of effect (Table 2). Studies that represent a low-level of evidence offer results that are likely to vary should the experiment be repeated, whereas a study offering a high-level of evidence provides greater confidence that the data are representative of valid results.

**Table 2:** Level of evidence by study quality score (Adapted from the GRADE Working Group, 2004 [35]).

Study quality score	Level of evidence	Confidence in estimation of effect
0–5	Very low	The estimation of effect is uncertain
6–10	Low	Further evidence is very likely to impact on our confidence in the estimation of effect and are likely to change the estimate
11–15	Moderate	Further evidence is likely to impact on our confidence in the estimation of effect and may change the estimate
16–20	High	Further evidence is very unlikely to change our confidence in the estimation of effect

A meta-analysis of results from comparable studies was not possible due to the heterogeneity between studies in terms of differences in participant samples (people with hearing loss, hearing aid users, and cochlear implant users), training stimuli, training protocols, and outcome measures adopted. As such, study findings and study quality were incorporated within a narrative synthesis to aid interpretation of findings and to examine any differences in outcomes across the 13 articles included in the systematic review.

## Results

Table 3 summarises the data extracted from each of the 13 articles. Where publications reported more than one study or training protocol these are presented separately in the table.

**Table 3:** Descriptive summary of extracted data from the 13 included articles.

Study	Design	Participants		Training			Outcomes	Main findings		Compliance and follow-up
		Hearing loss and hearing device	n, age and sex	Stimuli	Frequency and duration	Laboratory- or home-based training?	(bold indicates trained stimuli)	Trained stimuli	Untrained stimuli	
Fu et al. 2004 [36]	Repeated measures	7 pre-lingually and 3 post-lingually deafened adults. Existing cochlear implant users.	n = 10. 25–60 years, mean = 42.4 years. 4 male, 6 female.	Phonetic (vowel and consonant contrast) training with monosyllabic word, tailored to baseline performance.	1×1–2 hour sessions per day, 5 days per week, duration not reported.	Home	**Consonant and vowel recognition, v**oice gender discrimination, HINT sentences.	Improvements for vowel (14.4%***, 10/10 participants tested) and consonant (13.0%**, 7/10 tested) recognition. No improvement in voice gender recognition (0.3%, 7/10 tested).	Improvements in open set word-in-sentence recognition on HINT (27.9%**, 3/10 participants tested).	Compliance not reported. No follow-up assessment.
Burk et al. 2006 Experiment 2 [37]	Repeated measures	Mild to moderate bilateral SNHL.	n = 7. 65–75 years, mean = 69.6 years. 3 male, 4 female.	Right ear only, (open- or closed-set) monosyllabic AB words in noise (0 dB SNR), adjusted for audibility relative to individuals audiogram.	7×1 hour sessions in total over approx. 2 weeks (max. 3 days between sessions).	Laboratory	**Trained AB word recognition,** untrained AB word recognition.	Improvements in open-set (45.3%***) and closed-set (11.0%*) trained AB word recognition. No significant generalisation to trained AB words presented by untrained talkers.	Improvements in open-set untrained AB word recognition (6.9%*), no improvement for closed-set untrained AB word recognition (average improvement = 5.3%, *ns*). No significant generalisation to untrained AB words presented by a trained talker.	Compliance not reported. 5/7 participants returned 6 months post-training (Experiment 3). Performance remained significantly improved from baseline for trained open-set AB words (25.3%*), yet significantly worse than immediate post-training levels.
Burk et al. 2006 Experiment 3 [37]	Non-randomised controlled trial (control group = 9 young listeners, normally hearing.	Mild to moderate bilateral SNHL (5/7 participants from Experiment 2).	n = 5. 68–75 years, mean = 71.0 years. 2 male, 3 female.	Right ear only, (open- or closed-set) monosyllabic AB words in noise (0 dB SNR), adjusted for audibility relative to individuals audiogram.	Less than 1 hour (top-up training).	Laboratory	**Trained AB words recognition, trained AB words** in TIMIT sentences, untrained AB words in TIMIT sentences.	Trained AB words returned to within 95% critical difference of immediate post-training scores (Experiment 2). No significant improvements in trained AB words in TIMIT sentences.	No improvements in untrained AB words in TIMIT sentences.	Compliance not reported. No follow-up assessment.
Stecker et al. 2006 Experiment 1 [38]	Randomised controlled trial crossover (control group trained second).	Mild to moderate bilateral SNHL. New hearing aid users.	n = 23. 50–80 years, mean = 69.0 years. 23 male, 0 female. Immediate training (IT) n = 12. Delayed training (DT) n = 11.	Adaptive nonsense syllable (NST) identification in noise.	5×35–70 minute sessions per week for 8 weeks.	Home	**NST**, R-SPIN.	IT group: Improvement of 10.6%*** in NST identification. DT group: 8.8%*** improvement. Trained NST improvements were shown to generalise to untrained voices (p<.001). Significant improvement in performance for trained NST presented by untrained talkers***.	5 subjects from the IT group and 6 from the DT group were tested on the R-SPIN. Average improvement of 3.3% (*ns*) attributed to lack of power.	92.5% average compliance reported. Participants completed 29–44 days of training out of a required 40 days. Mean training days = 37, mode = 40. No significant decrement (−1.1%, *ns*) from post-training performance for NST syllable identification at an 8 week follow-up assessment.
Stecker et al. 2006 Experiment 2 [38]	Repeated measures	Mild-moderate bilateral SNHL. Existing hearing aid users.	n = 8. 61–75 years, mean = 67.7 years. Sex not reported.	Adaptive nonsense syllable (NST) identification in noise.	5×35–70 minute sessions per week for 8 weeks.	Home	**NST.**	Improvement (p<.01) in NST identification, although to a lesser degree than new hearing aid users in Experiment 1.	-	Compliance not reported. No significant decrease in performance for NST syllable identification at a 2 months post-training follow-up.
Sweetow and Henderson-Sabes, 2006 [39]	Randomised controlled trial crossover (control group trained second).	Normal hearing to severe SNHL. n = 56 existing hearing aid users.	n = 89. 28–85 years. Immediate training group (IT) n = 56, mean age = 63.2 years, sex not reported. Delayed training group (DT) n = 33, mean age = 64.2 years, sex not reported.	Listening and Communication Enhancement (LACE).	5×30 min sessions per week for 4 weeks.	Home	**LACE,** QuickSIN, HINT sentences, Listening Span, Stroop, HHIA/E, CSOA.	Improvements shown for all LACE measures overall: Speech/Babble, Time Compression, Competing Speaker, Auditory Memory and Missing Word (all p<.05). Non hearing aid users (n = 9) only improved significantly in Speech/Babble and Competing Speaker (p<.05).	Improvements in the trained group (Group 1, n = 38) for QuickSIN 45 dB (−2.2 dB SNR***) QuickSIN 70 dB (−1.5 dB SNR***), Listening Span (.5 sentences*), Stroop (7.5 points**), HHIA/E (7.5 points/17%**) and CSOA (.14**). No significant improvement in HINT (where the control group also showed improvement).	73% compliance (65/89 participants enrolled completed the training, immediate training group n = 38/56, delayed training group n = 27/33). Improvements are maintained for participants tested on QuickSIN and HINT (n = 42/65 tested), HHIA/E and CSOA (n = 31/65 tested) 1 month post-training. No statistical tests reported.
Burk and Humes, 2008 [40]	Repeated measures	Mild to moderate SNHL.	n = 8. 58–78 years, mean = 69.5 years. 4 male, 4 female.	Right ear only - Recognition of 75 lexically hard and 75 lexically easy CVC monosyllabic words (difficulty switched at training mid-point), presented in noise and adjusted for audibility relative to the audiogram.	20–24 sessions (average 3 per week), for approx. 12 weeks.	Laboratory	**Trained CVC word recognition,** untrained CVC word recognition, VAST sentences.	Improvement in exically hard trained words for open-set (47.4%***) and closed-set recognition (16.4%***). Lexically easy words also improved for both open-set (40.4%***) and closed-set recognition (17.2%***). Improvements for hard words (40.2%***) and easy words (35.0%***) when presented by unfamiliar talkers.	No improvements were shown for recognition of untrained words. Some individual but no group improvements on trained words embedded within VAST sentences.	Compliance not reported. Improvements in trained word recognition were maintained across weekly follow-up assessments beginning 7 weeks post-training and lasting for 7–8 weeks (no statistical tests reported).
Miller et al. 2008 [41]	Randomised controlled trial (hearing aid users: n = 8 trained, n = 4 controls. cochlear implant users: n = 8 trained, n = 8 controls).	Existing hearing aid users: n = 11 SNHL, n = 1 mixed loss at low frequencies. Existing cochlear implant users: n = 10 adult onset, n = 6 child onset deafness.	n = 28. n = 12 hearing aid users: 26–90 years, mean = 76.3 years.Sex not reported. n = 16 cochlear implant users: 35–81 years, mean = 55.5 years. Sex not reported.	Speech perception assessment and training system (SPATS): Syllable constituents and sentences (in quiet and in babble).	2×2 hour sessions per week for 6 weeks.	Laboratory	**SPATS,** HINT sentences, CST-AV, CST-A, CID sentences, W22 words, CNC.	Improvements in SPATS (nuclei and onset) in quiet and for hearing aid users (average 8% improvement), and for cochlear implant users (average 6% improvement). Improvement for pooled hearing aid and cochlear implant trained participants of 11% (average 7% improvement in quiet and 15% in noise), relative to controls.	Improvements in CST-AV, CID W22, CNC for hearing aid users (when HINT and CST-A data removed) of around 10%. Averaged over all measures, trained hearing aid users improved by 8% compared to controls. Improvements in all measures for cochlear implant users, with greatest gains for HINT in quiet. Overall, trained cochlear implant users improved by 13% relative to controls. Pooled trained participants improved on average 10% on untrained outcomes relative to controls. Statistical tests not presented for individual outcome measures.	Compliance not reported. No follow-up assessment.
Humes et al. 2009 [42]	Repeated measures	Older adults with hearing impairment n = 10 trained using protocol 1: High frequency pure-tone average (HfPTA) across 1, 2, 4 k Hz mean = 31 dB HL, SD 9.6. n = 6 trained using protocol 2: HfPTA mean = 26.4 dB HL, SD 14.1.	n = 16. Protocol 1: n = 10. Mean age = 70.2, SD = 6.8 years. 7 male, 3 female. Protocol 2: n = 6. Mean age = 72.8, SD = 7.6 years. 5 male, 1 female.	Closed set word identification training in noise (4 speakers). Protocol 1: repeated in blocks of 2400, Protocol 2: repeated in blocks of 600.	24×75–90 minute sessions over 8–12 weeks.	Laboratory	**Trained frequent word recognition,** CID sentences, VAST sentences, Frequent phrases.	Significant improvements shown for frequent word identification of around 18 rationalised arcsine unites (RAUs)*.	Significant improvements in CID sentences, VAST sentences and frequent phrases. Average improvements of between 12–20 RAUs*.	81% compliance reported (13/16 participants completed the requested training duration) across two training protocols. Non-complaint participants retained in main analyses. No follow-up assessment.
Stacey et al. 2010 [43]	Repeated measures	n = 2 pre-lingual, n = 10 post-lingual, n = 1 pre/post-lingually deafened adults. Existing cochlear implant users.	n = 11. 23–71 years, mean = 54.8 years. 6 male, 5 female.	2-AFC discrimination of words presented acoustically from options presented visually.	1 hour per day for 3 weeks.	Home	**Trained word and sentence (IEEE/SPIN) recognition,** IEEE sentences, BKB sentences, Consonant test, Vowel test, GBI.	No improvement in trained stimuli (trend for improvement in trained sentences identified with polynomial contrasts).	Improvement in consonant test (8.06%*).	73% compliance reported (8/11 participants completed the requested training duration). Three participants excluded having completed just five hours of the requested 15 hours training.No follow-up assessment.
Tyler et al. 2010 Experiment 1 [44]	Repeated measures	Post-lingually deafened adults. Existing cochlear implant users.	n = 3. 43–63 years, mean = 60.3 years. 1 male, 2 female.	8-loudspeaker presentation of sound localization and Spondee words in babble.	1–3 months. Details of training sessions not reported.	Home	**Spondee word recognition, Sound localization,** CNC, CUNY sentences, HINT sentences.	Improvement in sound localisation for subject 1 (reduction in RMS error of 7^o^***). Improvements in the adaptive spondee-in-noise test for subject 1 (p<.01– p<.001) and subject 2 (p<.001)	Improvement in HINT (32%***) for subject 1. Improvements in CNC in noise recognition (4%*) and HINT sentences (36%***) for subject 2.	Compliance not reported. Retention of improvements in sound localisation and HINT at 2 and 7 months post-training for subject 1, other participants not tested. No statistical tests reported.
Tyler et al. 2010 Experiment 2 [44]	Non-randomised controlled trial	Post-lingually deafened adults. Existing cochlear implant users.	n = 9. Trained: n = 3. 63–77 years, mean = 68.7 years. 1 male, 2 female. Controls: n = 6. Age not reported. 3 male, 3 female.	2-loudspeaker presentation of sound localization and Spondee words in babble.	1–3 months.	Home	**Sound localization, Spondee word recognition.**	Improvements in localization scores were shown for subjects 1 (p<.001) and 2 (p<.01). Significant improvements in recognition scores were shown for subject 1 (p<.05) and subject 3 (p<.05).	-	Compliance not reported. No follow-up assessment.
Oba et al. 2011 [45]	Repeated measures	Post-lingually deafened. Existing cochlear implant users.	n = 10. 46–78 years, mean = 66.4 years. 4 male, 6 female.	Sound Express: Digits-in-noise (speech babble and steady noise).	5×30 minute sessions per week for 4 weeks.	Home	**Digit-in-noise (babble),** HINT sentences, IEEE sentences.	Improvements in digits-in-noise (steady noise) SRT of −2.8** dB and digits-in-noise (babble) of −4 dB***.	Improvements for HINT sentences in babble (−2.9 dB SRT**) and IEEE sentences in steady noise (improvement in % correct of 1.5%*) and in babble (9.2%*).	100% compliance reported. Performance remained significantly improved from baseline at a 1 month post-training follow-up for all improved measures (p<.001).
Barcroft et al. 2011 [46]	Repeated measures	Mild-moderate bilateral SNHL. Existing hearing aid users.	n = 69. Multi-talker training group (MTG): n = 35,18 male, 17 female. Males: 18–87 years, mean = 67 years. Females: 23–89 years, mean = 62 years. Single-talker training group (STG): n = 34,21 male, 13 female. Males: 46–89 years, mean = 70 years. Females: 22–87 years, mean = 64 years.	I hear what you mean – listening comprehension activities in four-talker babble.	12×1 hour sessions, twice a week for 6 weeks.	Laboratory	Iowa consonant test, Build-a-sentence test, Four-choice discrimination test.	-	Improvement in four-choice discrimination test (13.7% points***), with scores for the single talker version significantly greater than those on the multi-talker version (p>.001). No effect of STG/MTG training group. Results from other outcomes not reported.	Compliance not reported. No follow-up assessment.
Ingvalson et al. 2012 [47]	Repeated measures	Post-lingually deafened. Existing cochlear implant users.	n = 5. 50–85 years, mean = 71.4 years. 2 male, 3 female.	Seeing and Hearing Speech program (vowel and consonant identification in words, sentences and phrases in multi-speaker babble).	4×1 hour sessions spread over four days.	Laboratory	**Vowel and consonant recognition in words, phrases and sentences,** SSQ, QuickSIN, HINT.	-	Improvement in HINT in quiet**, HINT at +15 dB SNR** and QuickSIN*.	Compliance not reported. Improvements in HINT and QuickSIN maintained at a 4-day post-training follow-up assessment.
Zhang et al. 2012 [48]	Repeated measures	Post-lingually deafened. Existing bimodal hearing aid and cochlear implant users.	n = 7. 51–78 years, mean = 64 years. 2 male, 5 female.	Sound Express: phoneme contrast training (vowels and consonants), for six subjects and monosyllabic word identification in multi-speaker babble for one subject.	1 hour per day, 5 days a week for 4 weeks.	Home	Vowel and consonant recognition, CNC words, AzBio sentences, Voice gender and emotion identification.	-	Improvements for six out of seven participants for speech recognition: Vowel (8.6%*) and consonant (9.8%*) identification, CNC words (14.9%*). No improvements were shown for AzBio sentences (8.3%, *ns*), nor for pitch-related performance (voicegender and emotion identification)	100% compliance reported. Improvements largely maintained at a one-month follow-up (vowel, consonant and CNC word recognition follow-up scores were significantly greater (p<.05) than baseline scores at the follow-up assessment).

Data for normally hearing participants are omitted from this table [42].

− = no data reported, *p<.05, **p<.01, ***p<.001. MSB = multi-speaker babble, SNHL = sensorineural hearing loss; SNR = signal to noise ratio, SRT = speech reception threshold, RAU = rationalised arcsine unit [49]; Adaptive spondee words in babble test [50]; Adaptive 12-choice spondee words with multiple jammers test [51]; AzBio sentences [52]; BKB = Bamford-Kowal-Bench sentence lists [53]; Build-a-sentence test [54]; CID Everyday Sentences = Central Institute for the Deaf everyday sentences [55]; CID W22 = Central Institute for the Deaf word lists [56]; CNC = consonant-vowel nucleus-consonant monosyllables [57]; CNC words [58]; Consonant recognition [59]; CSOA = Communication Scale for Older Adults [60]; CST-A = Connected Speech [audio] test [61]; CST-AV = Connected Speech [audio-visual] test [61]; CUNY = City University of New York sentences [62]; Everyday sounds localization test [63]; IEEE Sentences [64] Four-choice discrimination test [46]; GBI = Glasgow benefit inventory [65]; HHIA = Hearing handicap inventory for adults [66]; HHIE = Hearing handicap inventory for the elderly [67]; HINT = Hearing in noise test [68]; Iowa consonant test [69]; LACE = Listening and Communication Enhancement, [39]; Listening span test [11]; NST = Nonsense syllable test [70]; QuickSIN [70]; R-SPIN = Revised speech perception noise test [71]; SPATS = [72]; SPIN = Speech perception in noise test [73]; SSQ = Speech, Spatial and Qualities of Hearing Scale [74]; Stroop Color Word test [75]; TIMIT sentences [76]; VAST = Verb and sentence test [77]; Vowel recognition [78].

### Study Characteristics

Data extracted from the 13 articles are presented in terms of the PICOS criteria (*Participants, Intervention, Controls, Outcome measures, Study designs*).

#### Participants

Participant samples included people with hearing loss without hearing aids or cochlear implants [37], [40], [42], new [38] and existing hearing aid users [38], [41], [46], experienced cochlear implant users [36, 41 43–45, 47], and bimodal (cochlear implant plus hearing aid) users [48]. However, samples were not always consistent. Participants in the study by Burk et al. [37] were not all regular hearing aid users, and the participant sample in Sweetow and Henderson-Sabes [39] included nine participants who reported difficulty understanding speech in adverse listening environments, but did not use hearing aids. Participant sample sizes ranged from n = 3 [44] (Experiment 1) to n = 69 [46], with a median sample of 9.5 (mean = 17.75, SD = 20.33).

#### Intervention

Training stimuli, training frequency, and training duration varied substantially between studies. Several studies trained participants on small parts of speech, such as phonemes [36], monosyllabic words, vowels or consonants [37], [40], [42], [47], [48], spondee words [44], and nonsense syllables [38], [41]. Other studies trained participants using digits [45], words or sentences [41], [43]. The remaining studies trained their participants using hybrid communication training packages such as the Listening and Communication Enhancement (LACE) program [39], which included a variety of listening and cognitive tasks alongside interactive communication strategies, and ‘I hear what you mean’ [46], that comprised several listening comprehension tasks. Training sessions ranged from 30 minutes [39] to 2 hours per session [41], and occurred daily to twice-weekly. Training duration ranged from four days [47] to three months [44].

#### Controls

Ideally a systematic review would assess only high-level evidence arising from randomised controlled trials as randomisation, rather than between-group differences, substantially increases our confidence that any observed effects are attributable to the intervention. Nevertheless, inclusion of only randomised controlled trials in this review would have served to eliminate the majority of published evidence assessing the efficacy of auditory training for people with hearing loss. As such, other study designs were considered. However, only studies that reported direct comparisons between control and trained groups, or between control and trained periods within a subject group, were included in the review. Repeated measures design (where participants act as their own controls) was the most commonly identified study type.

#### Outcome measures

There were no outcome measures that were common to all training studies. The majority of studies assessed measures of speech intelligibility using validated speech tests such as the Hearing in Noise Test (HINT) [68], the Revised Speech in Noise test (R-SPIN) [71], IEEE sentences [64], and the Nonsense Syllables Test (NST) [70]. The study by Sweetow and Henderson-Sabes [39] also included behavioural measures of cognition (working memory: Listening Span [11], and attention: Stroop Task [75]) and self-report of hearing (either the Hearing Handicap Inventory for the Elderly (HHIE) [67] or the Hearing Handicap Inventory for Adults (HHIA) [67], and the Communication Scale for Older Adults (CSOA] [60]). The study by Ingvalson et al. [47] was the only other study to include a self-report measure of hearing (Speech and Spatial Qualities of Hearing Scale (SSQ) [74]), whereas Stacey et al. [43] assessed self-report of health status using the Glasgow Benefit Inventory (GBI) [65].

#### Study designs

Some articles reported more than one study. There were 11 repeated measures designs [36]–[38], [40], [42]–[48], two non-randomised controlled trials [37], [44] and three randomised controlled trials [38], [39], [41].

### On-task Learning

On-task learning was defined as any improvement in performance on a task or stimulus that had been directly trained. This was almost always reported in studies assessing the efficacy of auditory training for people with hearing loss (10/13). Nine articles reported significant on-task learning for trained stimuli. Despite trends towards improvement, Stacey et al. [43] did not show significant on-task learning for trained words in a group of 10 cochlear implant users. Barcroft et al. [46], Ingvalson et al. [47] and Zhang et al. [48] did not report any on-task learning results.

Burk and colleagues were the only authors to report multiple outcomes from variations in training protocols using the same word training stimuli. Humes et al. [42] reported significant and considerably smaller improvements (20%) in open-set word recognition for trained words presented in larger sets (600 words) than Burk and Humes, (40–55%) who presented words in smaller (150 words) sets [40].

### Generalisation of On-task Learning

Generalisation of learning was defined as an improvement in performance on a task or stimulus not directly trained. Outcomes that measured the generalisation of learning were divided into three sub-groups; improvements in speech intelligibility, cognition and self-report of communication.

#### Speech Intelligibility

All studies reported at least one measure of speech intelligibility. Table 4 provides a summary of these outcomes and any significant post-training improvement. Results are presented for untrained speech stimuli only, thus generalisation to improvements in performance for trained stimuli produced by different talkers is not considered here (refer to Table 3).

**Table 4:** Improvements in untrained measures of speech intelligibility by participant type.

	Study	Trainingstimulus	Outcome measures	Outcome stimulus	Laboratory- or home- based training?	Significant improvement?
**A.**	**People with hearing loss**					
	Burk et al. 2006 [37]	Monosyllabicwords	Word recognition	(SSN) Untrained monosyllabicAB words	Laboratory	**Y**
			TIMIT	(SSN) (Untrained AB) keywordsin sentences		N
	Burk and Humes, 2008 [40]	Monosyllabicwords	Word recognition	(SSN) Untrained monosyllabicCVC words	Laboratory	N
			VAST	(SSN) (Trained CVC) keywords in sentences		N
	Humes et al. 2009 [42]	Frequent words	CID Everyday Sentences	(ICRA 6) Sentences	Laboratory	**Y**
			VAST sentences	(ICRA 6) Sentences		**Y**
			Frequent phrases	(ICRA 6) Sentences		**Y**
**B.**	**Hearing aid users**					
	Stecker et al. 2006 [38], Experiment 1	Nonsensesyllables	R-SPIN	(MSB) Final keyword in sentences	Home	N
	Sweetow and Henderson Sabes, 2006 [39]	*LACE*	QuickSIN	(MSB) Sentences	Home	**Y**
			HINT	(SSN) Sentences		N
	Miller et al. 2008 [41]	*SPATS* syllables	HINT	(Q) and (SSN) Sentences	Laboratory	N
			CST-A	(Q) and (MSB) Sentences		N
			CST-AV	(Q) and (MSB) Sentences		N
			CID-W22	(Q) and (MSB) Words		N
			CNC	(Q) and (MSB) Monosyllables		N
	Barcroft et al. 2011 [46]	*I hear what you mean* listening comprehension	Four-choice discrimination test	(MSB) Words	Laboratory	**Y**
**C.**	**Cochlear implant users**					
	Fu et al. 2004 [36]	Phonemes	HINT	(SSN) Sentences	Home	**Y**
	Miller et al. 2008 [41]	*SPATS* syllables	HINT	(Q) and (SSN) Sentences	Laboratory	N
			CST-A	(Q) and (MSB) Sentences		N
			CST-AV	(Q) and (MSB) Sentences		N
			CID-W22	(Q) and (MSB) Words		N
			CNC	(Q) and (MSB) Monosyllables		**Y**
	Stacey et al. 2010 [43]	Words	Vowel test	(Q) h-vowel-d words	Home	N
			Consonant test	(Q) a-consonant-a nonsense words		**Y**
			BKB	(Q) Sentences		N
			IEEE	(Q) Sentences		N
	Tyler et al. 2010 [44] Experiment 1	Spondee words	CNC	(Q) CNC monosyllabic words	Home	N
			CUNY	(MSB) Sentences		N
			HINT	(SSN) Sentences		**Y**
	Oba et al. 2011	*Sound Express*digits	HINT	(SSN) and (MSB) Sentences	Home	**Y**
			IEEE	(SSN) and (MSB) Sentences		**Y**
	Ingvalson et al. 2012 [47]	*Seeing and* *Hearing Speech*monosyllabicwords	QuickSIN	(MSB) Sentences	Laboratory	**Y**
			HINT	(MSB) Sentences		**Y**
**D.**	**Bimodal (cochlear implant and hearing aid) users**					
	Zhang et al. 2012 [48]	*Sound Express*phonemesmonosyllabicwords	Vowel recognition	(MSB) h-vowel-d words	Home	**Y**
			Consonant recognition	(MSB) a-consonant-a nonsense words		**Y**
			CNC words	(MSB) Words		**Y**
			AzBio sentences	(MSB) Sentences		N

Significant improvement **Y** = yes, N = no. (Q) stimuli presented in quiet; (SSN) stimuli presented in speech-shaped noise; (ICRA 6) stimuli presented in ICRA (track 6) two-talker noise-vocoded competition; (MSB) stimuli presented in multi-speaker babble.

A number of studies reporting untrained speech intelligibility measures identified measures that had a degree of overlap between the lexical content of the trained and outcome stimuli. For example, Burk et al. [37] reported a 6–9% overlap and tested trained word recognition embedded in untrained sentences. Humes et al. [42] reported substantially greater overlap between the lexical content of trained and outcome stimuli of 50–80%. For some studies the degree of overlap was unclear, for example, the ‘Four Choice Discrimination Task’ reported by Barcroft et al. [46] appears to be very similar in nature to a trained exercise, although no details about any lexical overlap are provided.

Results revealed mixed findings for all participant groups, training stimuli, and study designs, whereby generalisation of learning to untrained measures of speech intelligibility did not always occur. For example, Burk et al. [37] reported that training on words generalised to improvements in untrained words and to trained words by untrained speakers, but did not generalise to trained words embedded in TIMIT [77] sentences. Similarly Zhang et al. [48] showed post-training improvements in the intelligibility of untrained vowels, consonants and words, but no improvements in performance for untrained sentences. Typically, where improvements were reported, the magnitude of improvement for people with hearing loss was small. For example, older hearing impaired listeners in Burk et al. [37] improved on untrained word recognition by an average of 6.9% compared to 45.3% in younger normally hearing listeners. Average improvement in untrained measures of speech intelligibility in people with hearing loss, ranged from 3.3% for sentences [38], to 14.9% average for words [48]. Sweetow and Henderson-Sabes, [39] were the only authors to report effect sizes for improvements in speech outcomes for people with hearing loss following training using LACE. Despite small reported effect sizes (*ES*) for an untrained measure of speech intelligibility, (QuickSIN: improvement of −1.5 dB signal to noise ratio (SNR) when presented at 70 dB, *ES* = 0.23, improvement of −2.2 dB SNR when presented at 45 dB, *ES* = 0.31) [70], the authors suggested that 46% of participants achieved clinically significant post-training improvements (defined as an improvement of −1.6 dB or greater in the SNR). No significant improvements were shown for HINT sentences, which the authors attributed to improvements also being shown in the control group, suggesting likely test-retest improvement effects.

#### Cognition

The study by Sweetow and Henderson-Sabes [39] was the only study to include cognitive outcome measures. Significant post-training improvements were shown for measures of attention (Stroop, 7.5 points) and working memory (Listening Span, 0.5 sentences). However, unlike the results for speech intelligibility measures in the same study, effect sizes were not presented for these cognitive outcomes. Furthermore, due to the hybrid (auditory-cognitive) nature of the training stimuli, it is not clear which element(s) of LACE contributed to the improvements in cognition.

#### Self-reported communication

Sweetow and Henderson-Sabes [39] demonstrated significant post-training improvements in self-reported hearing handicap using the HHIE and HHIA [67] and the CSOA [60], with effect sizes of around.4. However, Ingvalson et al. [47] did not report statistically significant post-training improvements for the SSQ [74].

### Retention of Learning

Retention of learning was defined as (i) the maintenance of a significant improvement from pre-training baseline performance, or (ii) a non-significant decrease in post-training performance, at a delayed post-training follow-up assessment. Follow-up assessments were reported in 8/13 articles, ranging from 4 days to 7 months post-training.

#### Retention of on-task learning

Burk et al. [37] reported that trained word-recognition performance at baseline (37.6%) was significantly improved six months post-training (62.9%, *p*<.05), but significantly worse than immediate post-training performance (83.5%, *p*<.05). The authors reported that participants were returned to peak post-training performance levels with as little as one hour of top-up training, although no additional follow-up was conducted to identify for how long peak performance was maintained. Stecker et al. [38] reported that Nonsense Syllable Test (NST) scores for new hearing aid users, showed no significant decrement from immediate post-training performance (9.8%) at an eight week follow-up (8.7%). For existing hearing aid users, the same was true, despite a smaller post-training improvement. Burk and Humes [40] tested participants on the same outcomes once a week for seven weeks after completion of two training protocols (easy and hard words), with no significant reduction in performance over the seven week period (Table 3). Tyler et al. [44] reported retention of trained sound localization and spondee-in-noise performance at two and seven months post-training, although no statistical tests are reported due to the small sample size. Finally, Oba et al. [45] reported retention of trained digit recognition performance at a one-month post-training that was significantly better than pre-training baseline, with no significant reduction from post-training performance levels.

#### Retention of generalised improvements in untrained outcomes

Sweetow and Henderson-Sabes [39] reported that at the time of publication, 65% (42/65) of trained participants had completed both QuickSIN and HINT sentences, and 48% (31/65) of participants had completed the HHIE, HHIA and the CSOA questionnaires, at a four-week follow-up. Post-training improvements were reported to be maintained for all measures, although no statistical analyses were presented. Tyler et al. [44] reported retention of improvements for HINT sentences seven months post-training for subject 1 (of 3) only. However, the authors also reported a gradual improvement in performance over time at pre-training assessments for subject 1, suggesting that some degree of post-training improvement in this measure may be attributable to test-retest improvement effects. Oba et al. [45] reported retention at one month post-training was significantly greater than pre-training baseline performance for both HINT and IEEE sentences (in steady noise and in babble) with no significant change between immediate post-training and follow-up performance. Ingvalson et al. [47] reported no significant performance differences four days post-training for HINT and QuickSIN sentences. Zhang et al. [48] reported maintenance of a significant increase from pre-training baseline at a one month post-training assessment, for measures of vowel, consonant and CNC word recognition. However, no information was provided as to whether performance on these measures was significantly reduced from immediate post-training levels.

### Compliance with training

Compliance was defined as the percentage of participants completing the requested training duration in each study. Compliance was reported in less than half of the articles (6/13) and the method of identifying those participants who did not achieve 100% compliance differed between studies. Stecker et al. [38] reported that on average, participants achieved 37 of the requested 44 days of training (92.5%). Sweetow and Henderson-Sabes [39] reported overall compliance of 73% (65/89 participants completed the training) although no details of training duration were provided for those who had completed. Humes et al. [42] reported that 13/16 participants (81%) completed the requested training duration. The remaining three participants completed 92%, 75% and 50% of the requested training. As these participants did not significantly differ in performance on the post-training outcomes compared with fully-compliant participants (CID Everyday Sentences [55], frequent words and phrases [42], and VAST sentences [78]), data from these low-compliant participants were included in the main analysis. In the article by Stacey et al. [43], compliance was 73% and three participants who completed only five of the requested 15 hours of training were excluded from the main analysis. Oba et al. [45] reported that 100% of participants achieved the required training duration of 600 minutes, despite reported training durations ranging from 583–767 minutes. Similarly, Zhang et al. [48] stated that 100% of participants completed the 20 hours requested training, but reported a mean training duration of 18 hours (range 15.4–21.2 hours).

### Quality Assessment

Study quality was assessed using five measures of scientific study validity, and five training-specific study quality criteria (each scoring 0–2), resulting in a possible maximum study quality score of 20. Table 5 shows individual study validity ratings and overall study quality scores for each of the 13 articles. Overall study quality ranged from very low (lowest score for Barcroft et al. [46], scoring 1/20) to moderate study quality (highest score for Sweetow and Henderson-Sabes, [39], scoring 13/20).

**Table 5:** Study validity criteria, study quality scores and levels of evidence for included articles.

Article	Scientific study validity criteria					Training-specific study validity criteria					Study quality score	Level of evidence^1^
	Randomisation	Control group	Power calculation	Blinding	Outcome measure reporting	Outcome measure selection^2^	Training feedback	Ecological validity	Reporting of compliance	Follow-up		
Fu et al. (2004) [36]	0	0	0	0	1	2	2	2	0	0	**7**	low
Burk et al. (2006) [37]	0	1	0	0	2	1	2	0	0	2	**8**	low
Stecker et al. (2006) [38]	1	2	0	0	1	1	2	2	1	2	**12**	moderate
Sweetow and Sabes, (2006) [39]	1	2	0	0	2	2	2	2	1	1	**13**	moderate
Burk and Humes (2008) [40]	0	0	0	0	2	2	2	0	0	1	**7**	low
Miller et al. (2008) [41]	0	2	0	0	2	2	0	0	0	0	**6**	low
Humes et al. (2009) [42]	0	0	0	0	2	2	2	0	2	0	**8**	low
Stacey et al. (2010) [43]	0	0	0	0	2	2	2	2	2	0	**10**	low
Tyler et al. (2010) [44]	0	1	0	0	1	1	2	1	0	1	**7**	low
Oba, Fu and Galvin, (2011) [45]	0	0	0	0	2	2	2	2	2	2	**12**	moderate
Barcroft et al. (2011) [46]	1	0	0	0	0	0	0	0	0	0	**1**	very low
Ingvalson et al. (2012) [47]	0	0	0	0	0	2	0	0	0	1	**3**	very low
Zhang et al. (2012) [48]	0	0	0	0	2	2	2	2	1	2	**11**	moderate

Criteria scoring: 0 = flawed or no information from which to make a judgement, 1 = weak information, incorrect use or lack of detail from which to make a judgement, 2 = appropriate use and reporting. Study quality score = sum of scores for scientific and training-specific study validity criteria. 1. Level of evidence: Study quality score of 0–5 = very low, 6–10 = low, 11–15 = moderate, 16–20 = high (adapted from GRADE Working Group, 2004 [35]). 2. Outcome measure selection: to assess generalisation of learning to untrained measure(s) of speech intelligibility, cognition or communication.

#### Scientific study quality

Out of a maximum 10 points for the scientific study quality, the highest scoring article achieved a total of five points [39]. None of the included articles reported participant or tester blinding, or a power calculation to determine the required participant sample size. Where participant randomisation was used, there was often a lack of detail on how randomisation was conducted [38], [39], [46]. However, more than half the articles (8/13) scored 2 points for the adequate reporting of all included outcome measures in their studies.

#### Training intervention-specific study quality

The majority of articles (10/13) reported the use of performance feedback in their training protocols. Reporting of participant compliance with training regimens occurred in almost half of the included articles (6/13). However, definitions of non-compliance varied. For example, some studies reported this as the number of participants remaining in the study irrespective of whether they had completed the requested amount of training, e.g. [39]. Others considered this to mean to completion of the requested training duration, e.g. [43]. Follow-up assessments were reported in 8/13 articles, and ranged from repeated testing of participants at weekly intervals [40] to single follow-up assessments [37]–[39], [44], [45], [47], [48]. Training in the participant’s home environment occurred in approximately half of the studies [36], [38], [39], [43]–[45], [48], while the remaining studies delivered training in the laboratory [37], [40]–[42], [46], [47]. Although the majority of studies (11/13) assessed and reported the generalisation of learning to untrained measures of speech intelligibility, some to cognition or communication, outcome measures were not always reported in adequate detail [37], [38], [44]. Furthermore, there were frequent reports of test administration inconsistencies whereby not all participants were administered all outcome measures [36], [38], [46], training stimuli varied between participants [48], and findings from some outcome measures were omitted from the results [46], [47].

#### Risk of funding bias

Sweetow and Henderson-Sabes [39] acknowledged a potential conflict of interest in funding. They reported a financial interest in Neurotone, Inc., the company licenced by the University of California, San Franscisco to produce LACE training software.

## Discussion

The primary aim of this systematic review was to examine the evidence for individual computer-based auditory training (CBAT) as an effective intervention for people with hearing loss. Secondary aims sought to examine the feasibility of individual CBAT as an intervention for people with hearing loss by examining (i) the long-term retention of auditory training-related improvements at post-training follow-ups, and (ii) levels of participant compliance with individual CBAT protocols.

### Efficacy of Individual Computer-based Auditory Training as an Intervention for People with Hearing Loss

Following a program of individual CBAT, significant improvements on the trained task (on-task learning) were shown for all but one of the articles that reported on-task outcomes [9/10]. However, evidence for the generalisation of learning to functional benefits (i.e. speech intelligibility) for people with hearing loss was mixed. A narrative synthesis and quality assessment of included articles suggested that evidence was not robust, and a number of confounding factors contributed to the inconsistency in reported effects. First and foremost, a lack of homogeneity in training protocols (training stimuli, duration or frequency), outcome measures, participant samples (sample size, hearing loss) and study designs may have resulted in variations in reported outcomes. Second, where generalisation of learning was shown to untrained measures, improvements were often highly variable between trained individuals [36], [42], [43], [48] and not everyone was shown to benefit from auditory training [39]–[41], [44], [45], [48]. Previous research into the neurophysiological changes resulting from auditory perceptual learning for normally hearing adults suggests that although the auditory system is responsive to training, there is a substantial degree of variability across individuals in their ability to make use of physiological cues [79].

In a previous review of the efficacy of auditory training for adults with hearing loss [18], the authors concluded there was little evidence for real-world effectiveness, but some evidence for within-study efficacy, of individual auditory training for people with hearing loss. A more recent meta-analysis of six (predominantly clinician-delivered) auditory training studies published between 1970 and 2009 [26] suggested reliable but small improvements in speech recognition (Cohen’s *d* = .352). Findings from the present review are similar in that on-task learning nearly always occurred for people with hearing loss following individual CBAT. Furthermore, there was some evidence for the generalisation of that on-task learning to untrained measures of speech intelligibility, cognition and self-reports of communication. However, the magnitude of improvement for untrained outcomes is small, and reported improvements are shown to be inconsistent across different studies, and within studies across individual trainees.

### Feasibility of Individual Computer-based Auditory Training as an Intervention for People with Hearing Loss

Feasibility was considered in terms of the retention of training-related improvements and compliance with individual CBAT. Although retention of post-training improvements was shown for a range of on-task and untrained measures at follow-up assessments up to 7 months post-training, the definition of retention varied across studies. The majority focused on the retention of improvements for trained tasks, not the retention of generalised improvements in untrained measures of speech intelligibility, cognition and communication. It is the latter that holds the most promise for individual CBAT to improve the everyday listening abilities of people with hearing loss.

Details of participant compliance with training were often underreported, appearing in less than half of the articles included in the review (6/13). Where reported, participant compliance rates were high. However, these reports of high compliance with training were not consistent with a large-scale study of more than 3000 clinical LACE trainees [80], where compliance (defined as completion of 10 or more training sessions) was around 30% [81]. This may suggest that rates of compliance with individual CBAT may be greater in smaller, controlled research-settings than may be typically expected in clinical environments.

### Study Quality and Evidence of Bias

Study quality scores suggest that the articles included in this review offer very-low to moderate levels of evidence. None of the studies reported participant or tester blinding. Thus, where between-groups designs are employed and the control group received no intervention [38], [39], [41], [44], we cannot be confident that any training improvements were not biased by placebo effects. The study by Sweetow and Henderson-Sabes [39] that obtained the highest quality score was the only study to report cognitive outcomes. Results showed significant generalisation of learning from trained stimuli (LACE) to untrained measures of speech intelligibility (QuickSIN), cognition (attention: Stroop Task, and working memory: listening span), and self-report of communication (HHIE, HHIA and CSOA).

The majority of studies failed to account for test-retest improvements in reported outcomes. When administering the outcomes across multiple test sessions, there is a possibility that improvement will occur as a result of procedural rather than perceptual learning [82], [83]. It has been recommended for the HINT and QuickSIN sentences that practice with at least two sentence lists is needed to eliminate procedural learning effects at baseline sessions [83]. Only two of the articles repeated outcome measure assessments at baseline sessions. Studies by Fu and colleagues [36] administered outcomes for a minimum of two weeks prior to training, and Stacey and colleagues [43] repeated baseline measures for approximately three hours per participant, both until a performance asymptote was reached. The total number of occasions outcomes that administered was not reported in either study. Finally, two articles omitted findings from some outcome measures included in the studies [46], [47]. Selective outcome reporting is likely to lead to inaccurate and misleading conclusions being reached [84].

### Lack of High-quality Evidence as a Barrier to implementation

Results from this systematic review demonstrate robust on-task learning following individual CBAT. Generalisation of on-task learning to functional benefits for people with hearing loss is less robust. Evidence for the generalisation of on-task learning to improvements in speech intelligibility, cognition and self-report of hearing suggests that improvements are both small and inconsistent across studies and individual trainees. Inconsistencies in reported effects may in part be associated with inconsistencies in study designs, training protocols, participant samples, and outcome measures adopted. However, analysis of study quality has demonstrated some fundamental issues with scientific control, which may result in a range of biases in reported effects. Nevertheless, retention of learning from both trained and untrained stimuli was shown to persist where reported, up to seven months post-training. Furthermore, where participant compliance was reported, rates were high. This suggests that individual CBAT has the potential to be a feasible intervention, which may offer benefit to the auditory-perceptual abilities of people with hearing loss.

Nevertheless, some of the questions posed by Boothroyd [85] in a discussion of the potential role of formal training in adapting to changed hearing, remain unanswered in the current evidence-base. First, where benefits occur, what are the mechanisms of benefit of auditory training for people with hearing loss (i.e. where generalisation of learning is shown, what elements of on-task learning are these attributable to)? How do individual characteristics interact with training outcomes? And, do training-induced changes influence participation and quality of life for people with hearing loss? Although many of these questions are currently being explored in normally hearing listeners [86]–[88] there is a need for further research designed to specifically address these issues in people with hearing loss.

### Recommendations for further research

Based on the reviewed evidence we propose key recommendations for future research:

#### 1. High-level evidence

High quality studies that are randomised, blinded, with a sample size dictated by a power calculation, are crucial to adequately assess the efficacy of individual computer-based auditory training for people with hearing loss. Furthermore, the possible inclusion of an ‘active’ control group (that is a task comparable to the training group task, but for which no improvement in performance is expected), may help enable participant blinding to help ensure any training-related improvements are not influenced by placebo effects. Future research would ideally be reported in accordance with the CONSORT statement [89], which offers guidelines for the reporting of randomised controlled trials. This would result in sufficiently detailed reporting to allow for an adequate appraisal of the quality and applicability of published results. It is also important that future studies consider key factors pertinent to training intervention studies, including ecologically valid training environments, performance related feedback, and follow-up assessments to ascertain the long-term benefits of auditory training and adequate reporting of participant (non-) compliance as interventions can only ever be beneficial if individuals comply with them.

#### 2. Outcome selection

Measures that are appropriate for and sensitive to CBAT effects should be adopted to allow accurate assessment of training efficacy. This includes a consideration of the magnitude of effect required for those outcomes to represent clinically significant improvements in listening abilities for people with hearing loss, for example, combining behavioural outcomes with questionnaires to assess self-reported benefits in everyday listening. In addition, the inclusion of cognitive outcomes in future studies assessing the efficacy of individual CBAT for people with hearing loss may be informative given that the only study to include such measures in this review reported significant post-training improvements in attention and auditory working memory [39]. Evidence from a study of LACE training with normally hearing adults suggested that training improves the neural representation of cues important for speech perception [86]. However, Tremblay and colleagues argue that at least that some of the physiological changes as a result of auditory training may not reflect sensory-specific fine-tuning, but other top-down modulatory influences that are activated during focused listening tasks, such as stimulus exposure, attention, memory, decision-making and task execution [88]. Thus, measurement of both auditory and cognitive outcomes may help to characterise the mechanisms of benefit for people with hearing loss following auditory training.

#### 3. Standardisation of outcome measures

Standardisation of outcomes across auditory training studies would enable comparisons to be made between different training protocols. Furthermore, common outcome measures would enable meta-analyses of data from future training intervention studies.

#### 4. Candidature

Published evidence suggests that post-training improvements in untrained outcomes are highly variable, and not everyone benefits from auditory training [39]–[41], [44], [45]. Thus, identification of those individuals most likely to benefit from auditory training would be of substantial clinical importance, enabling clinicians to individually target those for whom CBAT would be most effective, and consider alternative interventions for those who are least likely to benefit from training.

### Summary and Conclusions

Individual computer-based auditory training (CBAT) is a time and cost efficient, flexible self-management intervention that has the potential to be delivered to people with hearing loss in their home environment. It is easily accessible to the target population via PCs and the internet [27], and can be tailored to individual needs. The present review identifies scientific, methodological and study quality issues in each of the 13 articles included in this systematic review. Our findings demonstrate that although individual CBAT is a feasible intervention for people with hearing loss, published evidence for the efficacy of individual CBAT to improve speech intelligibility, cognition and hearing abilities for adults with hearing loss is neither consistent nor robust. As such, the evidence cannot be used reliably to guide intervention at this time. Future high level evidence and the standardisation of outcome measures across different training studies will provide an evidence-base from which to adequately assess the efficacy of auditory training as an intervention for people with hearing loss.

## Supporting Information

Checklist S1 **Preferred Reporting Items for Systematic Reviews and Meta-analyses (PRISMA) Checklist.**
(DOC)

Example Search Terms S1 **Example terms used to search the PubMed database.**
(DOCX)
