# Supplementary material for: Efficacy of Individual Computer-Based Auditory Training for People with Hearing Loss: A Systematic Review of the Evidence
Source: PLoS One. 2013 May 10;8(5):e62836. doi: 10.1371/journal.pone.0062836 (PMC3651281; doi:10.1371/journal.pone.0062836)
Supplement: Example Search Terms S1 — Example terms used to search the PubMed database. (DOCX) [file pone.0062836.s002.docx]

Hearing*[Title/Abstract] OR Cochlear[Title/Abstract]) AND ((perceptual learning[Title/Abstract]) OR (perceptual training[Title/Abstract]) OR (auditory training[Title/Abstract]) OR (auditory learning[Title/Abstract])) AND (Humans[Mesh] AND English[lang] AND adult[MeSH] AND ("1996/01/01"[PDat] : "2011/08/18"[PDat]))

Limits applied for:

Human subjects; English language; Adults; Date range: 01/01/1996-present (18/08/11)
